# Melatonin Relieves Ozone Stress in Grape Leaves by Inhibiting Ethylene Biosynthesis

**DOI:** 10.3389/fpls.2021.702874

**Published:** 2021-07-28

**Authors:** Chuang Liu, Hui Kang, Yafang Wang, Yuxin Yao, Zhen Gao, Yuanpeng Du

**Affiliations:** State Key Laboratory of Crop Biology, Collaborative Innovation Center of Fruit & Vegetable Quality and Efficient Production in Shandong, College of Horticulture Science and Engineering, Shandong Agricultural University, Tai-an, China

**Keywords:** grape leaves, melatonin, ozone stress, ethylene, antioxidant capacity

## Abstract

Ozone (O_3_) stress severely affects the normal growth of grape (*Vitis vinifera* L.) leaves. Melatonin (MT) plays a significant role in plant response to various abiotic stresses, but its role in O_3_ stress and related mechanisms are poorly understood. In order to understand the mechanism of MT in alleviate O_3_ stress in grape leaves, we perform a transcriptome analyses of grapes leaves under O_3_ stress with or without MT treatment. Transcriptome analysis showed that the processes of ethylene biosynthesis and signaling were clearly changed in “Cabernet Sauvignon” grapes under O_3_ and MT treatment. O_3_ stress induced the expression of genes related to ethylene biosynthesis and signal transduction, while MT treatment significantly inhibited the ethylene response mediated by O_3_ stress. Further experiments showed that both MT and aminoethoxyvinylglycine (AVG, an inhibitor of ethylene biosynthesis) enhanced the photosynthetic and antioxidant capacities of grape leaves under O_3_ stress, while ethephon inhibited those capacities. The combined treatment effect of MT and ethylene inhibitor was similar to that of MT alone. Exogenous MT reduced ethylene production in grape leaves under O_3_ stress, while ethephon and ethylene inhibitors had little effect on the MT content of grape leaves after O_3_ stress. However, overexpression of *VvACO2* (*1-aminocyclopropane-1-carboxylate oxidase2*) in grape leaves endogenously induced ethylene accumulation and aggravated O_3_ stress. Overexpression of the MT synthesis gene *VvASMT1* (*acetylserotonin methyltransferase1*) in tobacco (*Nicotiana tabacum* L.) alleviated O_3_ stress and reduced ethylene biosynthesis after O_3_ stress. In summary, MT can alleviate O_3_ stress in grape leaves by inhibiting ethylene biosynthesis.

## Introduction

Ozone (O_3_) in the troposphere is a highly oxidizing atmospheric pollutant. Elevated O_3_ concentration severely affects the growth and development of plants (Serengil et al., 2011), as well as human health (Karnosky et al., 2007; Borowiak, 2013). At present, the near-surface O_3_ concentration is increasing at an annual rate of 0.5–∼2.0% (Vingarzan, 2004) and is projected to increase by 40–60% at the end of the 21st century, when the tropospheric O_3_ concentration will reach 80 nL L^–1^ (Fiscus et al., 2005). O_3_ stress induces the release of large amounts of ethylene from leaf stomata, the damage of plant leaves caused by O_3_ is correlated with the release of ethylene (Tingey et al., 1976; Mehlhorn and Wellburn, 1987). As an important signal molecule, ethylene plays an important role in plant response to abiotic stress (Zhang M. et al., 2016). Ethylene biosynthesis begins with the formation of S-adenosyl-L-methionine (SAM) from methionine by SAM synthetase. Then, 1-aminocyclopropane-1-carboxylic acid (ACC) synthase (ACS) catalyzes SAM to produce ACC, and ACC oxidase (ACO) oxidizes ACC to ethylene (Najeeb et al., 2018).

Grapes (*Vitis vinifera* L.) are an important fruit crop grown worldwide. Previous investigation suggested that O_3_ stress adversely affects the photosynthetic system of grape (cultivar Cabernet Sauvignon) leaves, and exogenous MT treatment can alleviate O_3_ stress (Geng et al., 2016; Chen et al., 2020). Grapes and other plants have evolved various strategies to withstand abiotic stresses, such as regulating interactive hormone networks, including MT and ethylene (Arnao and Hernández-Ruiz, 2015; Ryu and Cho, 2015; Nguyen et al., 2016).

Melatonin is an indole derivative of tryptophan that is ubiquitous in plants and animals and has high efficiency, conservation, and strong antioxidant effects (Galano et al., 2011; Yang et al., 2018). MT synthesis in plants requires the participation of many enzymes, and the last step of the reaction is catalyzed by acetylserotonin o-methyltransferase (ASMT) (Kang et al., 2011). MT is an essential plant growth regulator, and both external application and endogenous induction can improve plant tolerance to drought, salinity, and other abiotic stresses (Zuo et al., 2014; Arnao and Hernández-Ruiz, 2015). For example, exogenous MT treatment can enhance the antioxidant capacity of cabbage (*Brassica oleracea* L.) (Zhang N. et al., 2016) and tea tree (*Camellia sinensis* L.) (Li et al., 2021) by increasing their anthocyanin content. In apple (*Malus domestica*), MT can regulate reactive oxygen species (ROS) signaling and activate the CBL1-CIPK23 (calcineurin B-like 1-interacting protein kinases23) pathway to regulate the expression of potassium channel protein genes, thereby improving salt tolerance (Li et al., 2016). Furthermore, exogenous MT can reduce the ion poisoning of mushrooms (*Agaricus campestris*) (Gao et al., 2020) and wheat (*Triticum aestivum* L.) (Al-Huqail et al., 2020). MT can also interact with other plant hormones [abscisic acid (ABA), jasmonic acid, salicylic acid, ethylene, etc.] to form a significant component of the plant immune system (Arnao and Hernández-Ruiz, 2018). For example, MT can increase GA (gibberellin) and reduce ABA content by regulating the expression of GA and ABA synthesis-related genes in cucumber seedlings and alleviating the inhibitory effect of a high salt environment on seedlings (Zhang et al., 2014). In *Arabidopsis thaliana*, MT reduces root meristem size by inhibiting auxin synthesis and polar transport (Wang Q. et al., 2016) and interacts with ethylene signaling pathways to improve disease resistance (Lee et al., 2014).

So far, the relationship between MT and other signaling molecules under abiotic stress is obscure, especially under O_3_ stress. Thus, this experiment has explored the key metabolic changes caused by increasing the MT content in grape leaves under O_3_ stress and its possible action mechanism. This research will promote the application of MT in improving the O_3_ tolerance of grapes and reveal the potential molecular mechanism of MT in regulating other signal molecules under O_3_ stress.

## Materials and Methods

### O_3_ Fumigation System

Two O_3_ fumigation systems were set up in the school vineyard (36°11′N, 117°06′E), which were divided into four parts, including open-top air chambers (OTCs), gas supply systems, O_3_ generation system, and O_3_ concentration monitoring system (Geng et al., 2017). The mainframe of the OTC is composed of galvanized steel pipes with a 3 cm diameter and is divided into two parts: the lower part is a regular octagonal prism with length and height of 1.1 and 2.2 m. The upper part is a regular octagonal pedestal; the area of the upper base of the pedestal is one in third of the area of the lower base, and the angle between the side and the vertical is 45°. The top is open to the atmosphere, and the sides are covered with particular polyethylene plastic film for the greenhouse; the outside is covered with a sunshade net. The installation height of the LED light source (LED cold light source plant light, SP501-N, 405 W, Shanghai Sanhao Electromechanical Co., Ltd.) is 1.5 m. To ensure the stability of the gas concentration in the OTC, the gap between the exhaust ports is gradually reduced from the center of the OTC to the four sides. The O_3_ generating system (WJ-HY5, Jinan Sankang) is a high-frequency O_3_ generator. The oxygen intake of the O_3_ generator can be modulated by adjusting the rotor flowmeter to control the O_3_ concentration. The O_3_ concentration monitor (DR70C-O_3_ type) in the OTC was used to measure the O_3_ concentration in real-time and transmits the data to the computer for observation and storage.

### Plant Materials, Growth Conditions, and Experimental Treatments

Two-year-old potted seedlings of grapevine cultivar “Cabernet Sauvignon” were used to explore the effects of exogenous MT and ethylene on grape leaves under O_3_ stress. Cuttings were planted in cylindrical pots with a diameter of 25 cm and a height of 35 cm (substrate:sand:soil = 2:1:1). The potted seedlings were cultivated in a greenhouse. When the new shoot leaves grew to 10–12 pieces, the plants with the same growth potential were selected and treated with water, 50 μM MT (Xu et al., 2019), 250 mg L^–1^ ethephon (Ma et al., 2021), or 2 μM aminoethoxyvinylglycine (AVG) (Xu et al., 2019) every 2 days at 6 p.m. (three times in total), and each treatment (1.5 L) was replicated in five plants. After that, the plants were exposed to 110 nL L^–1^ O_3_ for 3 h at 800 μmol m^–2^ s^–1^ light intensity at 8 a.m. (Geng et al., 2016, 2017).

All treatments were as follows: The roots were irrigated with clean water and the leaves sprayed with clean water without O_3_ treatment (control); the roots were irrigated with clean water, the leaves sprayed with clean water, and then plants were exposed to O_3_ (O_3_); The roots were irrigated with 50 μM MT, the leaves sprayed with water, and then plants were exposed to O_3_ (MT + O_3_); the leaves were sprayed with 250 mg L^–1^ ethephon, the roots irrigated with clear water, and then plants were exposed to O_3_ (Ethephon + O_3_); The leaves were sprayed with 2 μM AVG, the roots irrigated with clean water, and then plants were exposed to O_3_ (AVG + O_3_); the roots were irrigated with 50 μM MT, the leaves sprayed with 2 μM AVG, and then plants were exposed to O_3_ (MT + AVG + O_3_). After the treatment, leaves with similar nodes and sizes were selected for RNA-Seq analysis and determination of physiological indexes.

“Cabernet Sauvignon” tissue culture seedlings were used to evaluate the effect of increasing endogenous ethylene content on grape leaves under O_3_ stress. Healthy apical growth tips of “Cabernet Sauvignon” vines were removed in early summer to establish grapevine *in vitro* shoot cultures. The plant materials were sterilized (75% alcohol for 2 min, 4% sodium hypochlorite for 15 min) and cultured on MS medium supplemented with 30 g L^–1^ sucrose, 7.5 g L^–1^ agar powder, and 0.2 mg L^–1^ indole-3-butyric acid (IBA). The plants were kept in a growth chamber maintained at 25/20°C, with a photoperiod of 16 h light/8 h dark, and branches with at least one bud and leaf were used for subculture every month. Healthy 2-month-old seedlings with consistent growth were selected for infection treatment. The tissue culture bottle caps were opened one week before treatment adapt the seedlings to the external environment gradually.

### Chlorophyll Fluorescence Imaging and Determination of Related Enzyme Activities and Physiological Indexes

Rapid chlorophyll fluorescence imaging of grape leaves was performed using a fluorescence imaging system (PSI, Czechia). Hydrogen peroxide (H_2_O_2_) contents in leaves were estimated using the trichloroacetic acid (TCA) method at 390 nm (Velikova et al., 2000). Superoxide radical (O_2_^–^) was measured as described by Elstner and Heupel (1976) by monitoring the nitrite formation from hydroxylamine in the presence of⋅O_2_^–^. The tissue staining methods of H_2_O_2_ and O_2_^–^ were according to Thordal-Christensen et al. (1997) and Orozco-Cardenas and Ryan (1999), respectively. Reduced ascorbic acid (AsA) content was measured by bipyridine colorimetry, while reduced glutathione (GSH) was determined using 5,5′-Dithio-bis (2-nitrobenzoic acid) (DTNB) (Zhou et al., 2005). The total glutathione content was determined by the method of previous descripted (Kosugi and Kikugawa, 1985). Oxidized glutathione (GSSG) content was calculated by the difference between total glutathione content and GSH content, and then GSH/GSSG value was obtained. Determination of superoxide dismutase (SOD) was by photochemical reduction of nitroblue tetrazolium (NBT) (Giannopolitis and Ries, 1977). The SOD activity unit U was 50% inhibition of NBT photochemical reduction. Peroxidase (POD) activity was determined by monitoring the increase in absorbance at 470 nm, caused by guaiacol oxidation (Scebba et al., 2001). One unit of POD activity was defined as the change of A470 by 0.01 per min. The activity of catalase (CAT) was determined according to the method of Cakmak and Marschner (1992). CAT can decompose H_2_O_2_, and H_2_O_2_ has a strong absorption peak at 240 nm wavelength, reducing A_240_ by 0.1 per minute to a unit (U) of CAT enzyme activity. The chlorophyll content was measured by UV (ultraviolet) spectrophotometry (Yang et al., 2009), while the activity of ascorbate peroxidase (APX) was measured as previously reported (Nakano and Asada, 1981). One unit of activity for APX was defined as the amount of enzyme that degraded 1 μmol of AsA per min. The activities of glutathione reductase (GR), monodehydroascorbate reductase (MDHAR), and dehydroascorbate reductase (DHAR) were measured with a kit (Keming Biotechnology Co., Ltd., Suzhou, China). One unit of activity for GR was defined as catalyzing the oxidation of 1 nmol NADPH (nicotinamide adenine dinucleotide phosphate) per gram of sample per min. One unit of MDHAR activity was defined as 1 nmol NADH (nicotinamide adenine dinucleotide) oxidized per min per gram of sample. One unit of DHAR activity was defined as 1 nmol AsA produced per min per gram of sample.

### Determination of MT, Ethylene, and ACC Contents

The MT content was determined in reference to the previously reported method (Qianqian et al., 2015), with some modifications. The sample weight was 3.0 g, and the MT was extracted with analytical grade methanol; the final extract was purified by the C_18_ solid-phase extraction cartridge (ProElut^TM^, DIKMA, China) with the help of a vacuum pump, and then the volume was adjusted to 1 mL. The ethylene production rate was measured by gas chromatography (Shimadzu GC-16A, Japan) and repeated three times (Farmer et al., 1986). In order to avoid the wounding effect on ethylene production, the wound was wrapped in cotton with water after sampling and then sealed with a sealing film. The leaves were immediately put into a container and sealed, maintained in a light incubator at 25°C for 24 h, and then the gas was extracted into a 1 mL syringe for determination. The extraction and determination of ACC were according to Tucker et al. (2010). After O_3_ treatment, the samples were stored in liquid nitrogen.

### RNA-Seq and Quantitative Real-Time PCR

Transcriptome sequencing was conducted by OE Biotech Co., Ltd. (Shanghai, China). Total RNAs were extracted using TRIzol Reagent (Invitrogen, Carlsbad, CA, United States), and the mRNA was enriched using magnetic beads containing Oligo (dT). The quality of the constructed gene library was checked by the Agilent 2100 Bioanalyzer (Agilent Technologies, Santa Clara, CA, United States). After passing the quality test, the HiSeq X Ten sequencer of Illumina Company was used for sequencing, and the double-terminal data of 150 bp was produced. Raw data (raw reads) were processed using Trimmomatic (Bolger et al., 2014). The reads containing ploy-N and the low quality reads were removed to obtain the clean reads. Then the clean reads were mapped to reference genome^1^ using hisat2 (Kim et al., 2015). The reads were reassembled using StringTie (Pertea et al., 2015). The protein-coding gene expression was calculated in FPKM (Fragments Per kb Per Million Reads). The default screening difference condition was *P* < 0.05 and log_2_ (fold change) >1. FDR (false discovery rate) error control method was used for *P*-value multiple hypothesis testing and correction. GO (Gene Ontology) enrichment and KEGG (Kyoto Encyclopedia of Genes and Genomes) pathway enrichment analysis of differentially expressed genes (DEGs) were respectively performed using R based on the hypergeometric distribution. The quantitative real-time PCR (qRT-PCR) analysis was carried out with the UltraSYBR Mixture kit (CWBIO, Beijing, China) on a Bio-Rad iQ5 (Hercules, CA, United States) instrument. The reaction mixture was 20 μL: double distilled water (ddH_2_O) 7.0 μL, forward primer (10 μmol L^–1^) 1.0 μL, reverse primer (10 μmol L^–1^) 1.0 μL, 2 × UltraSYBR Mixture 10.0 μL, cDNA 1.0 μL. The *Vvactin* gene was used as an internal reference. The relative quantitative gene expression values were calculated using the 2^–Δ^ ^Δ^ ^CT^ method from three replicates. The primer sequences used are shown in Supplementary Table 1.

### Genetic Transformation of *ACO2* and *ASMT1* in Grape and Tobacco

The open reading frames (ORFs) of *ACO2* and *ASMT1* from “Cabernet Sauvignon” leaves were cloned and then respectively ligated to the pRI101-AN expression vector driven by the 35S promoter. Then, the plasmid was transformed into *Agrobacterium tumefaciens* strain GV3101 by the heat shock method. The “Cabernet Sauvignon” tissue culture seedlings were immersed in an *Agrobacterium* suspension adjusted to OD_600_ = 0.6, placed in a closed container, and then the bacteria solution was completely immersed in grape leaves (with obvious water stains) by vacuum extraction. The bacterial suspension on the leaf surfaces was dried, and the seedlings were cultured in bottles with medium. After 2 days, a qRT-PCR analysis was done to detect the expression level, and the overexpression strain was used for the O_3_ treatment experiment. The plants infected with an empty carrier were used as a control, and each line was set with three replicates. The tobacco was infected by the leaf disc method (Wang F. et al., 2016), and the T0 tobacco plants overexpressing *VvASMT1* were obtained after screening in selection medium. The transgenic lines were further identified by PCR and confirmed by qRT-PCR, after which T2 transgenic lines were obtained for experimental treatment.

### Statistical Analysis

All statistical analyses were performed by SPSS 24.0 software. A one-way analysis of variance (ANOVA) followed by Duncan’s multiple range test was employed, standard deviation (SD) was calculated from three replicates. The differences between individual means were deemed to be significant at *P* < 0.05.

## Results

### Exogenous MT Inhibits the Ethylene Biosynthesis and Signaling Caused by O_3_ Stress

**FIGURE 1:**
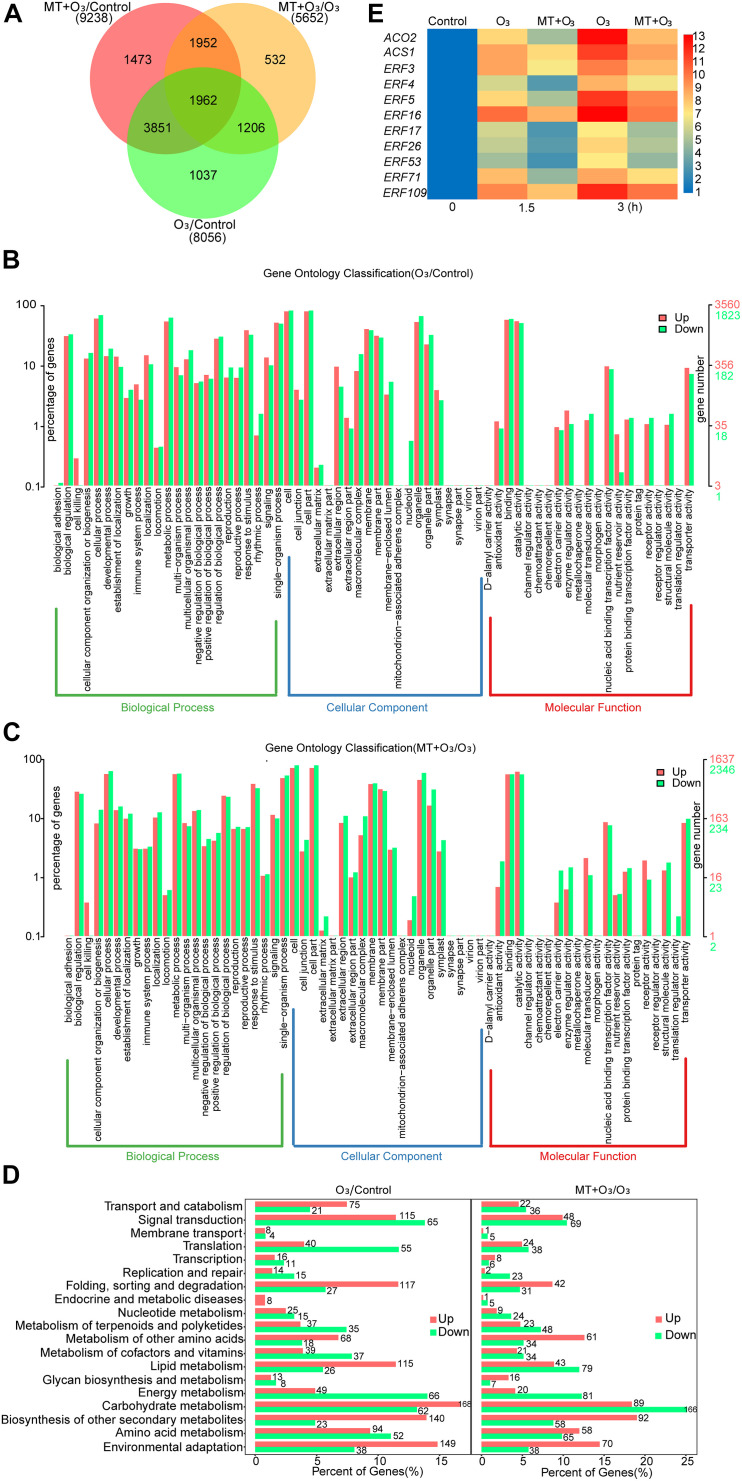
The number **(A)** and functional category **(B–D)** of DEGs (differentially expressed genes) and changes in the expression of genes related to ethylene biosynthesis and signal transduction **(C)** caused by O_3_ and MT + O_3_ treatment. **(A)** The number of common and unique DEGs between different comparison groups was counted to construct a Venn diagram. **(B,C)** GO (Gene Ontology) enrichment analysis of DEGs. **(D)** Pathway analyses of DEGs were carried out using the KEGG (Kyoto Encyclopedia of Genes and Genomes) database. **(E)** qRT-PCR was used to analyze the relative expression of genes related to ethylene biosynthesis and signal transduction after O_3_ and MT + O_3_ treatment for different times, and then a heat map was constructed. The normalized gene expression value was displayed as a color scale. MT, melatonin; ACS, 1-aminocyclopropane-1-carboxylate synthase; ACO, 1-aminocyclopropane-1-carboxylate oxidase; ERF, ethylene-responsive transcription factor.

To explore the mechanism by which MT alleviates O_3_ stress in grape leaves, RNA-Seq analysis was performed on “Cabernet Sauvignon” grape leaves treated with control, O_3_, and MT + O_3_. DEGs were represented by a Venn diagram (Figure 1A). Compared with the control, O_3_ significantly (*P-*value < 0.05) up-regulated and down-regulated 5121 and 2935 genes in grape leaves, respectively (Supplementary Table 3). Compared with O_3_ treatment, MT + O_3_ up-regulated and down-regulated 2342 and 3310 genes, respectively (Supplementary Table 4). GO enrichment analysis showed that the DEGs were primarily associated with biological regulation, cellular process, metabolic process, signaling, cell, cell part, membrane, membrane part, binding, catalytic activity, nucleic acid binding transcription factor activity, and transporter activity (Figures 1B,C). KEGG enrichment analysis indicated that all of the annotated DEGs were primarily related to signal transduction, amino acid metabolism, biosynthesis of secondary metabolites, carbohydrate metabolism, and environmental adaptation (Figure 1D). In the classification of signal transduction pathways, the most apparent change in the number of DEGs occurred in the ethylene signal pathway (Supplementary Tables 5, 6). Compared with the control, O_3_ resulted in significant changes in the expression levels of 71 genes related to ethylene biosynthesis and signaling pathways. Except for the obvious down-regulation of three genes, all the others were up-regulated; the expression of *ACO2* was up-regulated by 5.2-fold (Supplementary Table 5). Compared with O_3_ treatment, MT + O_3_ caused significant changes in the expression of 38 ethylene biosynthesis and signaling pathway-related genes. Among these 38 genes, 22 were significantly down-regulated, and *ACO2* was down-regulated by 1.03-folds (Supplementary Table 6). When the expression levels of these 22 genes in O_3_ treatment and control were compared, it was found that 19 had higher expression levels than the O_3_ treatment (Supplementary Table 7). qRT-PCR analysis was done to further determine changes in the expression of ethylene-related genes under O_3_ and MT + O_3_ treatments. The results showed that the expression levels of 11 genes increased significantly with the extension of O_3_ treatment time, among which the expressions of *ACO2* and *ERF16* (*ethylene-responsive transcription factor16*) were up-regulated by 12.7 and 12.9-folds, respectively (Figure 1E). During stress, the relative expression levels of all detected genes under the MT + O_3_ treatment were significantly lower than that under the O_3_ treatment (Figure 1E), which was similar to the transcriptome analysis results.

### MT Relieves O_3_ Stress by Regulating the Ethylene Pathway

**FIGURE 2:**
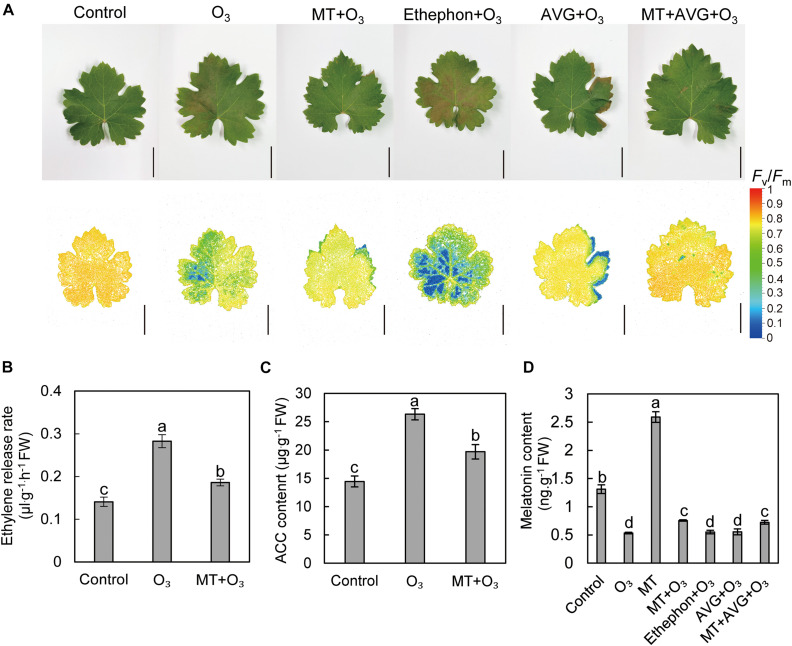
Effects of MT and ethylene on the apparent symptoms and chlorophyll fluorescence **(A)**, ethylene release rate **(B)**, ACC **(C)**, and MT **(D)** contents in grape leaves after O_3_ stress. AVG, aminoethoxyvinylglycine; ACC, 1-aminocyclopropane-1-carboxylic acid. Values represent the mean of three replicates ± SD. The difference was not significant at a 5% significance level among values labeled with the same lowercase letter. Bars, 5 cm.

To determine the effects of MT and ethylene on grape leaves under O_3_ stress, “Cabernet Sauvignon” grapes were treated differently. O_3_ stress caused more yellowing spots on grape leaves, and ethephon aggravated leaf damage symptoms under O_3_ stress, causing obvious chlorosis. In addition, AVG, MT, and a combination of the two treatments significantly reduced the leaf injury symptoms after O_3_ stress, and the yellowing area was smaller (Figure 2A). To further explore the mutual influence of MT and ethylene under O_3_ stress, the contents of MT and ethylene under different treatments were determined (Figures 2B–D). The results showed that compared with the control, O_3_ stress increased the ethylene release rate and the ACC content of “Cabernet Sauvignon” leaves by 100.8 and 82.19%, respectively. Meanwhile, the ethylene production after MT + O_3_ treatment was significantly less than in the O_3_ treatment (Figures 2B,C). Compared with the control, the MT content after O_3_ stress was significantly reduced by 59.25% (Figure 2D). After watering, the MT content in grape leaves increased by 97.64% relative to the control (Figure 2D). Compared with O_3_ treatment, the MT content after MT + O_3_ and MT + AVG + O_3_ treatment increased by 41.48 and 35.7%, respectively; however, there was no significant difference between ethephon + O_3_, AVG + O_3_, and the O_3_ treatment (Figure 2D).

### Effects of MT and Ethylene on F_*v*_/F_*m*_ and Reactive Oxygen Species in Grape Leaves After O_3_ Stress

**FIGURE 3:**
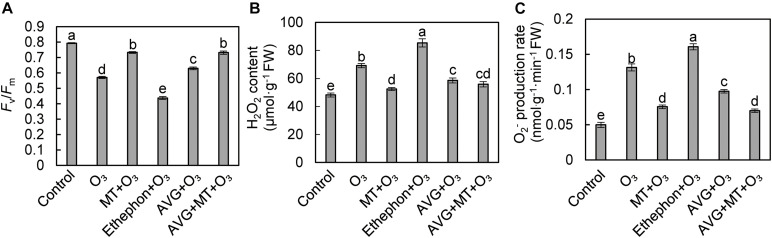
Effects of O_3_, MT + O_3_, Ethephon + O_3_, AVG + O_3_, and AVG + MT + O_3_ treatments on F_*v*_/F_*m*_ **(A)** and ROS **(B,C)** of “Cabernet Sauvignon” grape leaves. Values represent the mean of three replicates ± SD. Different lowercase letters above the bars indicate significant difference (*P* < 0.05).

Compared with the control, the F_*v*_/F_*m*_ of grape leaves after O_3_ stress decreased by 28.01% (Figure 3A). Compared with O_3_ treatment, the F_*v*_/F_*m*_ of grape leaves treated with ethephon + O_3_ decreased by 23.34%, while the F_*v*_/F_*m*_ increased by 28.35, 10.25, and 28.17% after MT + O_3_, AVG + O_3_, and MT + AVG + O_3_ treatment, respectively (Figure 3A). After O_3_ stress, the H_2_O_2_ content and O_2_^–^ production rate increased significantly by 43.55 and 163.30%, respectively, relative to the control. Compared with the O_3_ treatment, the H_2_O_2_ content and O_2_^–^ production rate of grape leaves increased by 23.37 and 22.30% after treatment with ethephon + O_3_, while MT + O_3_, AVG + O_3_, and AVG + MT + O_3_ decreased by 24.18 and 42.51, 15.31, and 25.78, 19.29, and 46.70%, respectively (Figures 3B,C).

### Effects of MT and Ethylene on Antioxidant System in Grape Leaves After O_3_ Stress

**FIGURE 4:**
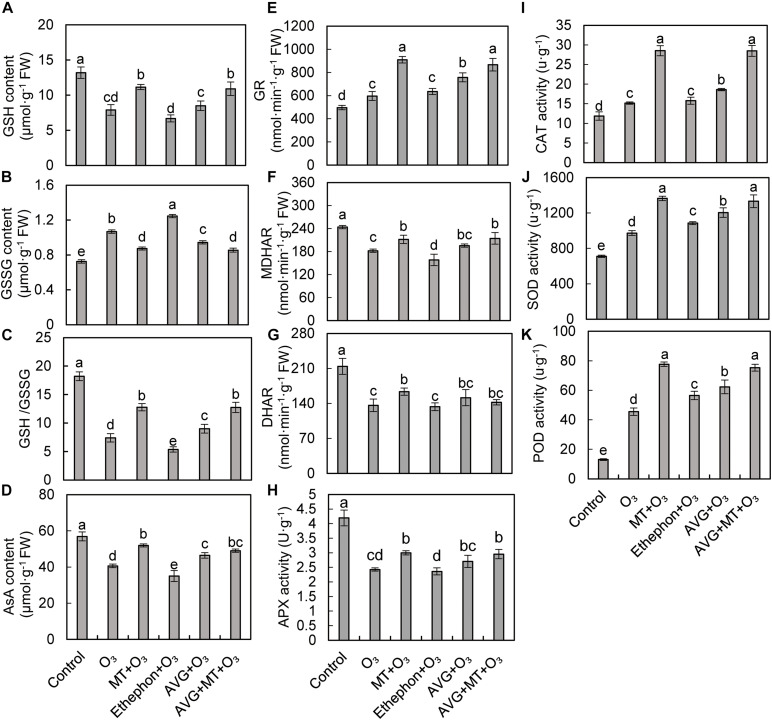
Effects of O_3_, MT + O_3_, Ethephon + O_3_, AVG + O_3_, and AVG + MT + O_3_ treatments on reduced glutathione (GSH, **A**) and oxidized glutathione (GSSG, **B**) contents, GSH/GSSG **(C)**, reduced ascorbic acid (AsA, **D**) content, glutathione reductase (GR, **E**), monodehydroascorbate reductase (MDHAR, **F**), dehydroascorbate reductase (DHAR, **G**), ascorbate peroxidase (APX, **H**), catalase (CAT, **I**), superoxide dismutase (SOD, **J**), and peroxidase (POD, **K**) activities in “Cabernet Sauvignon” grape leaves. Values represent the mean of three replicates ± SD. For the values labeled with the same lowercase letter, the difference was not significant, according to Duncan’s multiple range test at a 5% significance level.

Ascorbic acid and GSH are antioxidants involved in scavenging of active oxygen free radicals under stress conditions. Compared with the control, O_3_ stress significantly reduced GSH, AsA contents, and GSH/GSSG in grape leaves, while increased GSSG content (Figures 4A–D). Compared with O_3_ treatment, the GSH, AsA content, and GSH/GSSG were increased after MT + O_3_, AVG + O_3_, and AVG + MT + O_3_ treatments. However, ethephon + O_3_ treatment reduced GSH, AsA content, and GSH/GSSG, but increased the content of GSSG (Figures 4A–D).

Compared with the control, the GR, CAT, SOD, and POD activities in grape leaves increased significantly after O_3_ stress. In contrast, the activities of MDHAR, DHAR, and APX were significantly inhibited (Figures 4E–K). Compared with the O_3_ treatment, MT + O_3_, AVG + O_3_, and AVG + MT + O_3_ treatments increased the GR, MDHAR, DHAR, APX, CAT, SOD, and POD activities of grape leaves, but the difference between AVG + MT + O_3_ and MT + O_3_ treatment was not significant (Figures 4E–K). Compared with the O_3_ treatment, the SOD and POD activities of grape leaves after treatment with ethephon + O_3_ were significantly increased, the activities of MDHAR and APX were inhibited. In contrast, the activities of GR, CAT, and DHAR did not change (Figures 4E–K). The above results indicate that both MT and AVG can alleviate the damage caused by O_3_ stress on grape leaves by regulating the antioxidant system.

### Overexpression of *VvACO2* Intensifies O_3_ Stress in Grape Leaves

**FIGURE 5:**
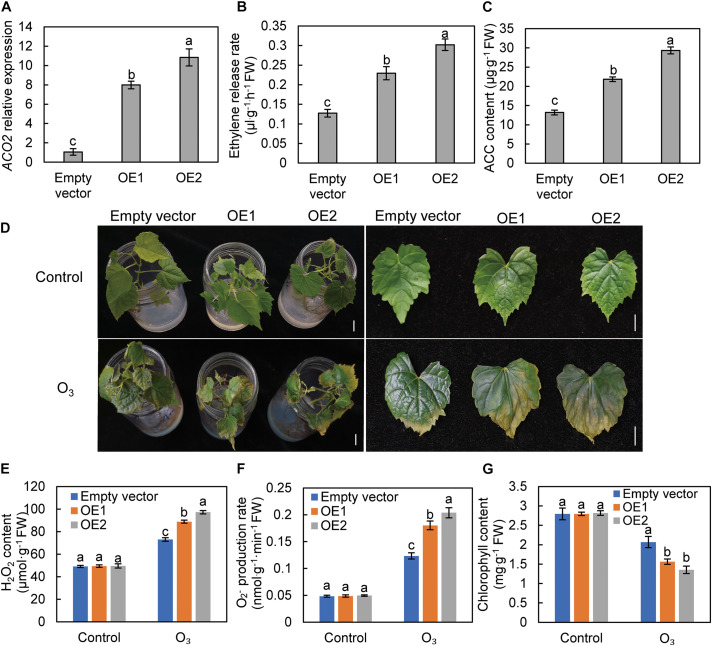
The effect of *VvACO2* overexpression on the response of grape leaves to O_3_ stress. The *ACO2* relative expression **(A)**, ethylene release rate **(B)**, and ACC content **(C)** in over-expressing (OE) and control (empty vector). The leaf appearance **(D)**, ROS **(E,F)**, and chlorophyll content **(G)** of the tissue culture seedlings of “Cabernet Sauvignon” grape after O_3_ stress. Letters marked by the same lowercase letters are not significant at *P* < 0.05 (Duncan’s multiple range test). Bars, 1 cm.

To further verify that ethylene can exacerbate the stress effect of O_3_ on grape leaves, the *VvACO2* gene was transiently overexpressed in grape leaves to promote ethylene biosynthesis. The expression levels of the two plants infected with 35S: *VvACO2* carrier were 7.98 and 10.83-folds that of plants infected with the empty carrier, respectively (Figure 5A). These results confirm the successful expression of *VvACO2* in grape leaves. The ethylene release rate and ACC content were also significantly higher in the leaves of plants overexpressing *VvACO2* than those of the control group (Figures 5B,C). Under normal conditions, the growth of the control and overexpression plants was the same. After 110 nL L^–1^ O_3_ treatment for 3 h, the yellowing degree (Figure 5D), H_2_O_2_ content (Figure 5E), and O_2_^–^ production rate (Figure 5F) in the leaves of overexpressed plants were significantly higher than those of the control group. Meanwhile, the chlorophyll content (Figure 5G) of overexpressed plant leaves was significantly lower than that of the control group. These results show that the endogenous induction of ethylene biosynthesis in grape leaves aggravated O_3_ damage to the leaves.

### Overexpression of *VvASMT1* Enhances Tobacco Tolerance to O_3_

**FIGURE 6:**
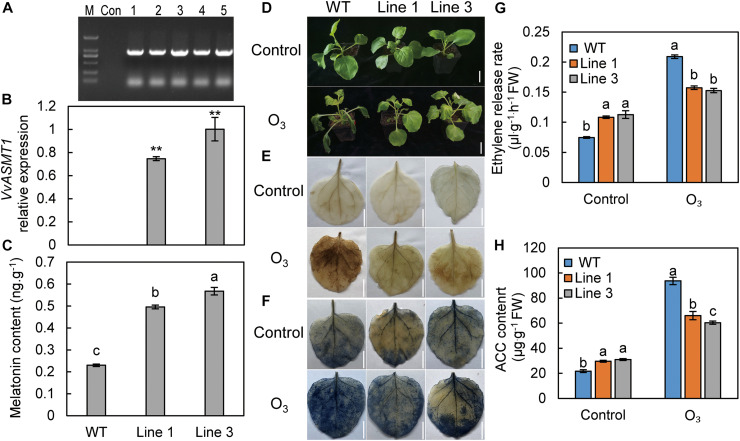
Tobacco plants overexpressing *VvASMT1* showed increased tolerance to O_3_. **(A)** PCR detection of *VvASMT1* overexpression in tobacco. **(B)** The expression level of *VvASMT1* in transgenic and wild-type tobacco. **(C)** The content of MT in transgenic and wild-type tobacco. Phenotypes **(D)**, H_2_O_2_ staining **(E)**, O_2_^–^ staining **(F)**, ethylene release rate **(G)**, and ACC content **(H)** of wild-type and transgenic lines after O_3_ treatment. The numbers 1–5 represent five different tobacco lines; M, DNA marker; Con, control; *ASMT*, *acetylserotonin methyltransferase*; WT, wild-type. Values represent the means ± SD of three replicates. **Highly significant difference, *P* < 0.01. Values indicated by the same lowercase letters are not significant at *P* < 0.05. Bars, 3 cm in **(D)**, 2 cm in **(E,F)**.

To analyze the effect of endogenous MT on plant O_3_ tolerance, the overexpression vector 35S: *VvASMT1* was transformed into tobacco to increase the endogenous MT content and test its O_3_ resistance. The upstream primer of the 35S promoter and the downstream primer for amplifying the *ASMT* gene were used for PCR amplification. The results showed no specific band in the control group, while specific bands appeared in the five tobacco lines. The band sizes were the sum of the 35S promoter fragment length and the *ASMT* ORF sequence length (Figure 6A). The results indicated that *VvASMT1* was successfully transferred into tobacco. Two lines with moderate expression and high expression, line 1 and line 3, respectively, were selected for functional analysis (Figure 6B). The results showed that the MT content in the leaves of the wild-type and the two transgenic tobacco lines were 0.2299, 0.4957, and 0.5676 ng g^–1^, respectively (Figure 6C). After treatment with 110 nL L^–1^ O_3_ for 3 h, the tobacco showed yellowing and wilting symptoms, and the stress degree of transgenic tobacco was significantly lower than that of wild-type tobacco (Figure 6D). The leaf color in the transgenic lines after H_2_O_2_ and O_2_^–^ staining was significantly lighter than that of the wild-type (Figures 6E,F), indicating that the ROS content was significantly lower than that of the wild-type. Compared with the wild-type, overexpression of *VvASMT1* significantly increased GSH, AsA content, and related antioxidant enzymes (GR, SOD, POD, CAT, MDHAR, DHAR, and APX) activities after stress (Supplementary Figure 1). After O_3_ treatment, the ethylene release rate and ACC content of transgenic tobacco leaves were significantly lower than the wild-type (Figures 6G,H). The above results indicate that overexpression of the *VvASMT1* gene in tobacco increased the MT content, alleviated the O_3_ stress, and reduced the ethylene content after stress.

## Discussion

At present, only a few studies have examined the molecular mechanism of O_3_ stress on fruit trees, especially grapes (Valletta et al., 2016). O_3_ enters plant leaves mainly through the stomata and increases ROS production (Langebartels et al., 2002). Thus, ROS level can be an essential indicator to determine the degree of cellular oxidative stress (Collén et al., 2003). When large amounts of ROS are produced under stress, its clearance system gets damaged, resulting in membrane lipid peroxidation, massive chlorophyll degradation, hindrance of photosynthetic electron transfer, inactivation of antioxidant enzymes, and inhibiting plant growth development (Degl’Innocenti et al., 2007; Iriti and Faoro, 2008; Bose et al., 2014). PSII is the most sensitive component of the photosynthetic electron transport chain under O_3_ stress (Tran et al., 2013). Similarly, our research showed that O_3_ stress could damage grape PSII and significantly reduce the chlorophyll content in grape leaves. Stressed leaves also produced large amounts of ROS and changed the activity of antioxidant enzymes, resulting in yellowing and wilting of grape leaves. Further, O_3_ stress significantly induced the upregulation of genes related to ethylene biosynthesis and some ethylene responsive transcription factors, as well as the increase of ethylene release rate and ACC content. In our study, increase in exogenous and/or endogenous ethylene content aggravated O_3_ stress. Therefore, it was speculated that O_3_ destroys the photosynthetic and antioxidant grapes systems by increasing the ethylene content.

Many studies have shown that MT can alleviate various abiotic stresses; plants with higher MT content are more tolerant to O_3_ (Dubbels et al., 1995). The present study also proved that MT could relieve O_3_ stress in grape leaves and improve various physiological indexes. Exogenous MT application significantly increased the F_*v*_/F_*m*_ of “Cabernet Sauvignon” grape leaves under O_3_ stress and alleviated leaf chlorosis. Similarly, MT can reduce the damage of barley leaf photosystem II (PSII) under stress and maintain the chlorophyll content (Arnao and Hernández-Ruiz, 2009). These results may be related to the protection of chloroplast structure by MT (Zhao et al., 2016). Additionally, this experiment found that watering the roots of “Cabernet Sauvignon” with MT could increase the leaf MT content. Similarly, treating grapes with MT at the rhizosphere increased the MT levels of the roots, but also in the leaves, thus enhancing salt tolerance in “Crimson seedless” grapevines (Xu et al., 2019). This result suggests that the MT provided through external sources is absorbed in plants (Tan et al., 2007) and can accumulate in distant organs through long-distance transportation to tolerate abiotic stress. In addition, our experiment for the first time verified that overexpression of *VvASMT* in tobacco could alleviate O_3_ stress by increasing the MT content. This result is similar to that obtained following overexpression of the key enzyme gene caffeic acid-O-methyltransferase (*COMT*) for MT synthesis, which increased the endogenous MT content of tomatoes and enhanced salt resistance (Sun et al., 2019).

Plants can resist ROS damage through a defense system composed of enzymatic and non-enzymatic ROS scavenging systems (Apel and Hirt, 2004). Antioxidant enzyme scavenging systems mainly include SOD, POD, CAT, etc., while non-enzymatic systems include the AsA-GSH cycle. GSH can catalyze the degradation of excess H_2_O_2_ and activate various defense mechanisms by participating in redox signal transduction (Szalai et al., 2009). The increase in GR activity reduces the cellular glutathione pool, providing sufficient GSH for DHAR to reduce dehydroascorbate (DHA) to AsA (Noctor and Foyer, 1998). MT can enhance the scavenging ability of the ROS system to improve stress tolerance. For example, the application of 0.5 μmol L^–1^ MT can improve salt tolerance in tomatoes by increasing the antioxidant enzyme activity and the accumulation of AsA and GSH (Liu et al., 2015). Moreover, the leaves of O_3_-tolerant soybean varieties maintain higher AsA levels than susceptible varieties (Chutteang et al., 2015). Thus, varieties with high antioxidant contents in the leaves are more resistant to the O_3_ damage. This experiment also found that MT could protect antioxidant enzymes and the AsA-GSH signaling system under O_3_ stress, while ethylene had the opposite effect. MT may act as an antioxidant to antagonize ethylene and remove excessive ROS, thereby sharing the pressure of other antioxidants.

Melatonin has opposite regulatory effects on ethylene in different crop species. For example, MT can promote the ripening of grape berries by increasing the ethylene content (Xu et al., 2018) and enhancing the salt tolerance of grapes by promoting *VviMYB108A*-mediated ethylene biosynthesis (Xu et al., 2019). On the contrary, MT treatment reduces ethylene release and improves fruit quality by inhibiting the expression of genes related to ethylene biosynthesis during apple storage (Onik et al., 2020). In our experiment, MT treatment of O_3_ stressed plants down-regulated the expression of ethylene biosynthesis and signal transduction genes in grape leaves. MT treatment also reduced the ethylene release rate and ACC content in O_3_ stressed leaves, thus alleviating O_3_ stress. The increase of endogenous MT in tobacco also reduced ethylene biosynthesis after stress and alleviated O_3_ stress. However, MT treatment of ‘Crimson seedless’ grapevines roots increased the ethylene release rate of leaves (Xu et al., 2019). These contrasting effects of MT may be due to the multi-pathway characteristics of MT synthesis, making it functionally specific in the developmental stage, tissues, and organs. In addition, the physiological effects of MT are pleiotropic (Byeon and Back, 2014; Zhang et al., 2014), and its regulation of ethylene could be indirect. The different inducing effects could also change the mutual regulation with other hormones; thus, the specific mechanism needs to be further explored. Although both MT and ethylene inhibitor significantly alleviated O_3_ stress in grapes, the effect of MT treatment was better than treatment with ethylene inhibitor treatment. In addition, the combined treatment effect of MT and ethylene inhibitor was similar to that of MT alone, and the treatment of ethylene and ethylene inhibitor did not affect the MT content under O_3_ stress. It can be seen that MT and ethylene may play upstream and downstream roles, respectively, in the signal pathway under O_3_ stress.

**FIGURE 7:**
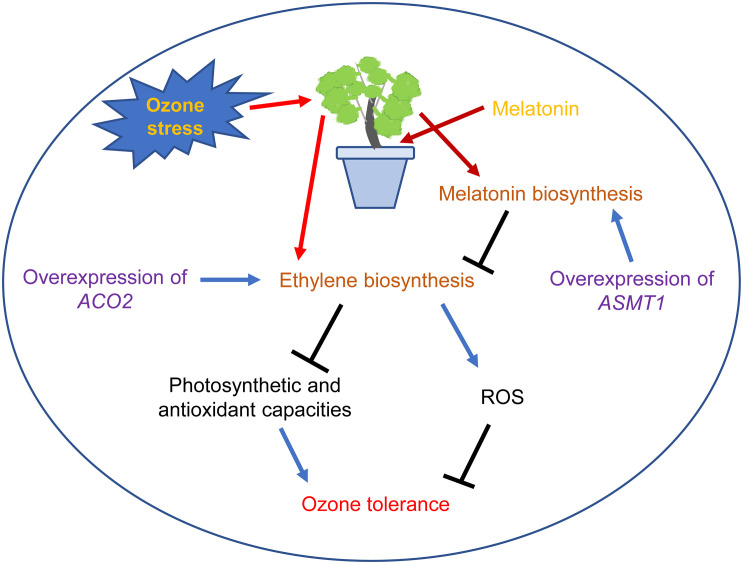
A schematic model showing the mechanisms of MT alleviating O_3_ stress in grape leaves. The arrow denotes increases and bar denotes decreases.

Finally, as depicted in Figure 7, O_3_ stress increased ROS content and decreased the photosynthetic and antioxidant capacities of grape leaves by inducing ethylene biosynthesis. Melatonin pretreatment or overexpression of *ASMT1* can enhance the *in vivo* melatonin level, reduce ethylene production in grape leaves under O_3_ stress and increase plant O_3_ tolerance. In addition, overexpression of *ACO2* in grape leaves decreased O_3_ tolerance by increasing endogenous ethylene content. Taken together, MT can alleviate O_3_ damage to grape leaves by inhibiting ethylene biosynthesis.

## Data Availability Statement

The original contributions presented in the study are publicly available. This data can be found here: NCBI repository, accession number: PRJNA733572.

## Author Contributions

CL performed most parts of the experiment, analyzed the data, and wrote the manuscript. HK and YW participated in performing the experiments. ZG participated in the manuscript writing and revison. YD and YY designed the research. All authors have read and approved the final manuscript.

## Conflict of Interest

The authors declare that the research was conducted in the absence of any commercial or financial relationships that could be construed as a potential conflict of interest.

## Publisher’s Note

All claims expressed in this article are solely those of the authors and do not necessarily represent those of their affiliated organizations, or those of the publisher, the editors and the reviewers. Any product that may be evaluated in this article, or claim that may be made by its manufacturer, is not guaranteed or endorsed by the publisher.

## Footnotes

1
http://genomes.cribi.unipd.it/grape/

## Supplementary Material

Supplementary Figure 1 Effects of ozone stress on GSH **(A)**, AsA **(B)**, and related antioxidant enzyme activities **(C–I)** of wild-type and transgenic tobacco.

Supplementary Table 1 Primers used in this study.

Supplementary Table 2 RNA-Seq profiles of grape leaves after different treatments, including control, O_3_, and MT + O_3_. The default condition for screening differences is *P* < 0.05 and fold change >2.
