# Supplementary material for: Melatonin Relieves Ozone Stress in Grape Leaves by Inhibiting Ethylene Biosynthesis
Source: Front Plant Sci. 2021 Jul 28;12:702874. doi: 10.3389/fpls.2021.702874 (PMC8355546; doi:10.3389/fpls.2021.702874)
Supplement: Supplementary Figure 1 — Effects of ozone stress on GSH (A), AsA (B), and related antioxidant enzyme activities (C–I) of wild-type and transgenic tobacco. [file Data_Sheet_1.docx]

**Supplementary data**

**Table S1.** Primers used in this study

| Gene name | Forward primers (**5ʹ-3ʹ**) | Reverse primer (**5ʹ-3ʹ**) | Gene ID and Purpose |
| --- | --- | --- | --- |
| *VvACO2* | TCCACACCAAACACCACAAAC | CACCAACTCAAAGAAACCCCA | VIT_212s0059g01380 qRT-PCR |
| *VvACS1* | ACCGTCTTTTCTCTCTCGCC | TCTTGGACAACAACTGCGGT | VIT_215s0046g02220 qRT-PCR |
| *VvERF3* | TCGGAGTTGTTTGGAAAGGG | TCAAATCCACCGCATCCAAC | VIT_207s0141g00690 qRT-PCR |
| *VvERF4* | CGAAACAGATGACGAAGCAA | TCAGTCGCAAAAAGGAGGTC | VIT_219s0014g02240 qRT-PCR |
| *VvERF5* | CCCGAAACTCCATGAAGATG | AAGTGTCAGAAATGGGTGGC | VIT_216s0013g01110 qRT-PCR |
| *VvERF16* | GCAGGTCCAGTTCCAAATGA | AGTGGCTATCACCGCATCTCTA | VIT_211s0016g00670 qRT-PCR |
| *VvERF17* | CGGTGGACTCGGGATTTCTC | AATCAGAGGGCGATGGCACA | VIT_204s0008g02230 qRT-PCR |
| *VvERF26* | GACTCAAGTCCCAACCCTCC | CTGGCAGTTCTGCTTCTCCA | VIT_216s0100g00400 qRT-PCR |
| *VvERF53* | TTTCCCAAAGCTCATCCCTC | TATCCCTAGTTTCCCCCACA | VIT_212s0059g00280 qRT-PCR |
| *VvERF71* | TCTTCTCCGCACAATCAACT | GGCCCAAATCGTATTCACAG | VIT_207s0005g00820 qRT-PCR |
| *VvERF109* | ACCCTGTTGTCACTCCCTCC | TAGCATCCTCCGTTTCACCC | VIT_207s0031g00710 qRT-PCR |
| *VvASMT* | AAGAAAGTGCGGACGAAGAG | TCCGTTAAATCTGGGTCGAG | LOC100243765 qRT-PCR |
| *Vvactin* | TCAGGAAGGACCTCTATGGC | CTGTGGACAATGGATGGACC | qRT-PCR |
| *VvASMT* | GTCGACATGGATTTGGCAAATGG | GGATCCAGGATAAACCTCAATAAGAGA | Overexpression |
| *VvACO2* | GTCGACATGGAGGCCTTCCCTGTGA | GGATCCGGCTGTTGCAATTGGCCC | Overexpression |
| *35S* | GGGTGCATCATCATCTTCTGTTG | GAAGACGTGGTTTTAACG | Verified overexpression |

Restriction Enzyme cutting sites are underlined


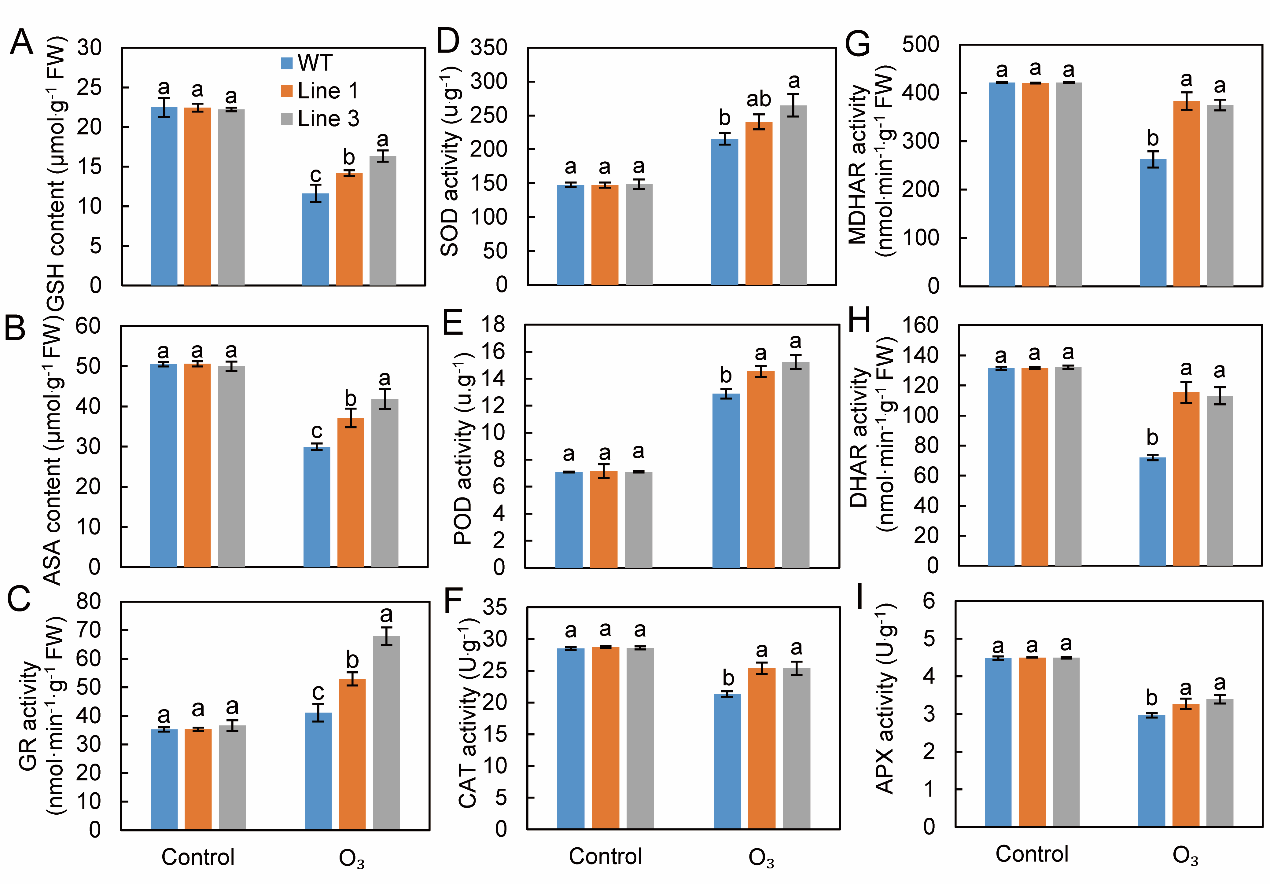


**FIGURE S1** Effects of ozone stress on GSH(**A**), AsA(**B**) and related antioxidant enzyme activities (**C-I**) of wild-type and transgenic tobacco.
